# Deciphering the structural consequences of R83 and R152 methylation on DNA polymerase β using molecular modeling

**DOI:** 10.1371/journal.pone.0318614

**Published:** 2025-03-12

**Authors:** Amit Srivastava, Haitham Idriss, Gobind Das, Sufian Abedrabbo, Mohd Sahir Shamsir, Dirar Homouz

**Affiliations:** 1 Department of Physics, Khalifa University of Science and Technology, Abu Dhabi, United Arab Emirates; 2 School of Public Health, Imperial College of Science, Technology and Medicine, London, UK; 3 Palestinian Neuroscience Initiative, Al-Quds University, Jerusalem, Palestine; 4 Faculty of Health Sciences, Global University, Beirut, Lebanon; 5 Department of Bioscience, Faculty of Science, Bioinformatics Research Group, Universiti Teknologi Malaysia, Johor Bahru, Malaysia; 6 Department of Physics, University of Houston, Houston, Texas, United States of America; 7 Center for Theoretical Biological Physics, Rice University, Houston, Texas, United States of America; Saveetha University - Poonamallee Campus: SIMATS Deemed University, INDIA

## Abstract

DNA polymerase β, a member of the X-family of DNA polymerases, undergoes complex regulations both in vitro and in vivo through various posttranslational modifications, including phosphorylation and methylation. The impact of these modifications varies depending on the specific amino acid undergoing alterations. In vitro, methylation of DNA polymerase β with the enzyme protein arginine methyltransferase 6 (PRMT6) at R83 and R152 enhances polymerase activity by improving DNA binding and processivity. Although these studies have shown that methylation improves DNA binding, the underlying mechanism of enhancement of polymerase activity in terms of structure and dynamics remains poorly understood. To address this gap, we modeled the methylated enzyme/DNA complex and conducted a microsecond-long simulation in the presence of Mg ions. Our results revealed significant structural changes induced by methylating both R83 and R152 sites in the enzyme. Specifically, these changes caused the DNA fragment to move closer to the C- and N-subdomains, forming additional hydrogen bonds. Furthermore, the cross-correlation map demonstrated that methylation enhanced long-range correlations within the domains/subdomains of DNA polymerase β, along with an increase in the linear mutual information value between the domains/subdomains and DNA fragments. The graph connectivity network also illustrated that methylation modulates the information pathway and identifies residues exhibiting long-distance coupling with the methylated sites. Our results provide an atomic-level understanding of the structural transition induced by methylation, shedding light on the mechanisms underlying the methylation-induced enhancement of activity in DNA polymerase β.

## 1. Introduction

The preservation of genome integrity is crucial for the survival and reproduction of organisms. DNA, being constantly exposed to various biological, chemical, and physical agents, is susceptible to different types of damage, including errors during replication [1–3]. To counterpart these threats, cells possess diverse DNA repair mechanisms such as Base Excision Repair (BER) [4], Nucleotide Excision Repair (NER) [5], Double Strand Break Repair (DSBR) [6], Homologous Recombination Repair (HRR) [7], and Non-Homologous End Joining (NHEJ) [8]. These repair mechanisms play a pivotal role in preventing the occurrence of cancer and neurogenerative diseases resulting from permanent DNA damage.

BER is a DNA repair mechanism designed to correct small damage to individual nitrogenous bases in DNA or breaks that typically do not distort the double helix [4]. This encompasses damages arising from various chemical base modifications or lesions [such as 8-oxo-7, 8-dihydro-2’-deoxyguanosine (8-oxoG), uracil, and alkylated bases]. BER is also involved in repairing the spontaneous loss of purine/pyrimidine bases from nucleotides in DNA strands, leading to the formation of apurinic/apyrimidinic (AP) sites [9,10]. BER can engage in short patch (one nucleotide long) or long patch (2-10 nucleotide gap) damage repair [4]. The BER process involves several key steps: Recognition and removal of the damaged base, Incision of the DNA strand, Resynthesis of the DNA strand, and Ligation of the nicked strand. Various proteins participate in BER, including DNA glycosylases, AP endonucleases, DNA polymerases, and ligases. DNA glycosylases identify and eliminate the damaged base, while AP endonucleases generate a single-stranded break at the damaged site. DNA polymerases fill in the gap with the correct nucleotide, and ligases facilitate the rejoining of the two strands, completing the repair process.

Structural data from crystallographic studies reveal a general pathway for nucleotide insertion [11–17]. Initially, DNA polymerase binds to DNA, forming an open binary complex, which then associates with the correct dNTP to create an open ternary complex. This structure transitions into a closed ternary complex, enabling nucleotide incorporation at the primer’s 3′-terminus. After the reaction, the complex reopens and releases pyrophosphate (PPi) as a by-product. These conformational shifts between open and closed states are essential for maintaining fidelity, ensuring accurate nucleotide selection via an “induced-fit” mechanism [18–20].

Eukaryotic cells express a variety of DNA polymerases, several of which participate in DNA repair mechanisms [21]. Among these is DNA polymerase β (pol β), a member of the X family of DNA polymerases. These enzymes are known for their compact size (pol β- 39kDa, 325 residues) and proficiency in filling short gaps in DNA [22]. Mutations in the gene encoding pol β have been associated with an increased risk of certain types of cancers, while impaired pol β are functionally linked to age-related disorders such as Alzheimer’s disease [21]. Pol β consists of two major domains: a 31 kDa domain exhibiting DNA polymerase activity and an 8 kDa domain that binds single-stranded DNA (ssDNA) and possesses deoxyribose phosphate (dRP-lyase activity). This enzyme facilitates the formation of a phosphodiester bond between the incoming nucleotide and the 3’-hydroxyl group of the preceding nucleotide in the growing DNA strand (polymerase function), while also excising the 5’ sugar phosphate moiety (lyase function). Structural studies have revealed that the active site of pol β contains a template strand-binding pocket and a catalytic pocket capable of accommodating incoming nucleotides [23]. The polymerase domain of pol β consists of three sub-domains: D, responsible for DNA binding; C, featuring the catalytic pocket; and N, binding the nucleotide triphosphate. These subdomains correspond to the thumb, palm, and finger subdomains, respectively, of DNA polymerase. A comprehensive understanding of the structure and function of pol β [24] is crucial for the development of new therapeutic approaches for diseases such as cancer and Alzheimer’s.

Pol β undergoes intricate regulation both in vitro and in vivo through diverse posttranslational modifications (PTM), including phosphorylation, acetylation, and methylation. The impact of these modifications varies based on the organism and the specific amino acid residue undergoing post-translational alterations. In mammalian pol β, in vitro phosphorylation by PKC at S44 and S55 [25,26] results in the inhibition of DNA polymerase function, while the ssDNA binding capability is maintained. Notably, phosphorylation at S44 alone has been suggested as sufficient to disrupt the polymerase function of pol β, and the role of adding phosphate at S55 remains unclear. Furthermore, acetylation of mammalian pol β at K72 [27] by the transcription activator p300 compromises the dRP-lyase activity of the enzyme. Interestingly, arginine methylation of pol β augments the polymerase function without impacting dRP-lyase activity. These differential effects underscore the nuanced and context-dependent regulatory mechanisms governing pol β functionally across different organisms and modification sites. Additionally, ligand and drugs may bind differentially to unmodified and posttranslationally modified enzymes which may be important for precision drug design.

Protein methylation [28,29], a PTM involving the addition of a methyl group (-CH_3_) to amino acid residues, is orchestrated by a family of enzymes termed protein methyltransferases. These enzymes facilitate the transfer of a methyl group from S-adenosylmethionine (SAM) to the side chain of the targeted protein’s amino acid. The process is reversible, with demethylases capable of removing the methyl group from the protein. Various amino acids, including lysine, arginine, and histidine, can undergo methylation. This modification plays a pivotal role in regulating protein function and has implications for the development of diverse diseases, including cancer. Pol β undergoes methylation in vitro on R83 and R152 by the enzyme protein arginine methyltransferase 6 (PRMT6). This process significantly enhances DNA polymerase activity by improving DNA binding and processivity, although it does not impact single nucleotide insertion and dRP-lyase activity. In vivo, the methylated pol β appears to be influenced by genotoxic stress.

Previous studies using molecular dynamics (MD) simulations have investigated the fidelity and conformational changes observed in kinetic experimental studies of DNA polymerase β [30,31]. Limited attention has been paid to the effect of mutation on the subdomain motions and free energy landscape of DNA polymerase β [32]. However, the impact of PTM on the subdomain motions and the structural transition of DNA polymerase β complex has not been explored yet. The precise mechanism by which PTM impacts the function of pol β remains unclear, leaving questions about whether the observed functional changes arise from structural alterations or simply result from a charge effect. Obtaining a crystal structure of the modified enzyme would elucidate this mechanism, but the complexity of achieving this experimentally necessitates alternative approaches. In our previous study [33,34], we successfully modeled the phosphorylated variant of pol β and proposed that the polymerase function may be attributed to the breakage of the hydrogen bond at Glu335. To delve deeper into understanding how methylation of pol β enhances the polymerase function, further information is required.

In this study, we modeled structures for the pol β methylated variants R83, R152, and R83, R152 together. The methylated arginine was modeled using the symmetric-dimethylarginine patch. We performed a microsecond-long simulation to study the structural change of methylated DNA pol β ternary complex in the presence of the ions. Our primary objective here is to study how the methylated arginine induces the structural transition in DNA pol β and affects the motions of its subdomain. Additionally, we looked at how the methylation changes the information pathway of DNA pol β. Our findings explain the underlying mechanism of the structural transition observed in DNA pol β due to the methylation and suggest that methylated arginine may upregulate the enzyme’s activity by inducing structural changes.

## 2. Methods

In this study, we choose the closed form of DNA pol β ternary complex that was taken from a protein data bank (PDBID: 2FMS) [35]. The system is composed of protein, DNA, and ions. To explore the conformational changes and dynamics of DNA polymerase β due to the methylation, the following systems were investigated: (1) methylated DNA polymerase β complex, where Arginine-83 (R83) was replaced by methylated residue, designated as meR83, (2) methylated DNA polymerase β complex, where Arginine-152 (R152) was replaced by methylated residue, designated as meR152, and (C) methylated DNA polymerase β complex, where both Arginine-83 (R83) and Arginine-152 (R152) was replaced by methylated residue, designated as meR83,152. The methylated arginine was modeled using the symmetric-dimethylarginine patch. The modeled structure included all the ions that were present in the crystal structure. The Mg ions were placed at the same positions as resolved in the crystal structure while Na and Cl ions were added randomly to neutralize the system. To explain the underlying mechanism of methylation-induced structural transitions in DNA polymerase β, we have simulated the above systems for 1 *μ* s. To study the role of DNA in methylated-induced transitions, we have simulated the systems in the absence and presence of DNA.

### 2.1. General MD simulation setup

MD simulations were conducted using the GROMACS 5.0.5 suite [36] under a constant temperature and pressure (NPT) ensemble. The CHARMM36 force field [37] was employed to characterize both the protein and DNA, alongside the TIP3P water model [38]. The system was solvated in a cubic box, ensuring a minimum distance of 10 Å from the box boundaries, with periodic boundary conditions applied in all directions. The equation of motion was integrated with a time step of 2 fs. The temperature was maintained at 300 K using the Berendsen thermostat, and the pressure was controlled isotropically at 1 bar using the Parrinello-Rahman barostat [39]. Neighbor searching was performed with a cutoff radius of 10 Å, and the non-bonded pair list was updated every 40 steps. Short-ranged interactions were truncated beyond 10 Å, with dispersion corrections applied. Long-range electrostatic interactions were calculated using the Particle Mesh Ewald (PME) method [40] with a grid spacing of 1.6 Å and interpolation of order 4. Using the SETTLE [41] and LINCS [42] algorithms, covalent bonds were constrained to their equilibrium geometries. The steepest decent method was used to minimize the energy of the system. The energy was minimized for 10,000 steps to remove unfavorable contacts from water and ion placement. After energy minimization, the systems were equilibrated in the NVT ensemble at 300 K with positional restraints for 10 ns to ensure stability, followed by another 10 ns of equilibration under the NPT ensemble at 1 bar and 300 K. The production run was performed for 1 *μ* s (1000 ns) for each system under unrestrained NPT conditions at 300 K and 1 bar. The coordinates are recorded every 2 ps for subsequent analysis.

For the structural analysis of the systems, we employed various built-in GROMACS functions [36], including gmx rms, gmx gyrate, gmx rmsf, and gmx hbond, on the trajectories generated for each DNA pol β complex system after MD simulations. These functions were used to calculate the root mean square deviation (RMSD), radius of gyration (R_g_), root mean square fluctuations (RMSF), and the number of hydrogen bonds (H-bonds). The criteria for H-bonds were set as a donor-acceptor distance d ≤3.5 Å, and the angle between the donor and acceptor is >30^0^. Matplotlib v3.3.2 [43] was used to plotting the results whereas, the structural images were produced using visual molecular dynamics (VMD) [44] and PyMOL [45]. Fig S1 (in the supplementary information) shows the flow chart of the MD simulation.

### 2.2. Principal component analysis

Principal component analysis (PCA) is a mathematical technique used to reduce the dimensionality of large datasets by transforming a large number of variables into a smaller set while preserving the original information. In the context of molecular dynamics (MD) simulations, PCA, also known as essential dynamics [46], is employed to analyze information about the direction of motions of the protein residues from a set of conformations produced by MD simulation. PCA works by diagonalizing the covariance matrix, where the elements are represented by $M_{ij}$. This covariance matrix is constructed from the Cartesian coordinates of the structures obtained from the MD simulations:


$$M_{ij}=\langle \left(r_{i}-\langle r_{i}\rangle \right)\left(r_{j}-\langle r_{j}\rangle \right)\rangle ,$$ (1)

where *i* and j represent all possible pairs of 3N Cartesian coordinates, where N is the number of atoms. In this work, the PCA was performed using $C_{\alpha }$ atoms of the protein and phosphate atoms of DNA. The covariance matrix, constructed from the atomic fluctuations of both the protein and DNA, was diagonalized. The resulting eigenvalues and eigenvectors from PCA can be used to describe the motion of the system. The first 20 eigenvectors were examined to access their cosine content. Of these, the two eigenvectors designated as PC1 and PC2, with cosine content less than or equal to 0.1, were used. These principal components were utilized to generate the free energy landscape (FEL) using the gmx sham function in GROMACS [36], which computes the minimum free energy configuration based on PC1 and PC2. The FEL map was subsequently plotted using MATLAB [47].

### 2.3. Perturbation response scanning

Perturbation response scanning (PRS) analysis [48,49] was conducted to identify the most critical and sensitive residues of methylated DNA polymerase β, using the anisotropic network model (ANM). In the ANM approach [50], the protein is represented as a network of nodes corresponding to the positions of $C_{\alpha }$ atoms for each residue, with inter-residue interactions modeled by harmonic springs within a threshold distance of 12 Å. PRS, based on linear response theory, evaluates the impact of perturbing individual residues by measuring changes in the protein’s dynamics. In PRS, each residue in the network is sequentially subjected to a force of fixed magnitude, and the overall network response is calculated using Hooke’s law (ΔR = H^−1^F), where F is the applied force and H is the Hessian matrix obtained from ANM. The displacement of all residues, ΔR^(*i*)^, is computed for each residue under the applied force, where i ranges from 1 to N. The PRS analysis focuses on the first 20 slowest non-zero eigenvalues of the ANM modes, which characterize the most energetically favorable collective motions in the protein structure. This method allows for the identification of key residues in DNA polymerase β.

## 3. Results and discussion

**Fig 1:**
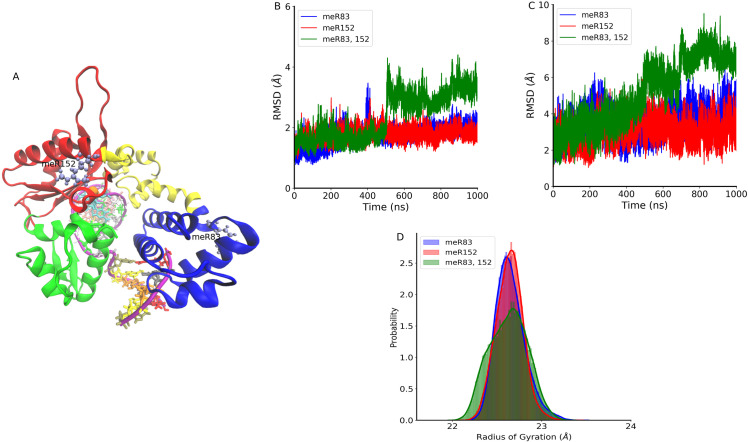
(A) Schematic representation of DNA polymerase β complex. The protein structure includes the Lyase domain (residues 10-87, blue color) and three subdomains: DNA binding (D, residues 90-150, orange color), Catalytic (C, residues 151-260, red color), and Nascent base pairing (N, residues 261-335, green color). These subdomains correspond to the thumb, palm, and fingers subdomains, respectively. The DNA is shown in purple color. The methylated residues (meR83, and meR152) are shown in CPK representation. (**B**) The time evolution of root mean square deviations (RMSD) of $C_{\alpha }$ atoms with respect to the initial structure of meR83 (blue), meR152 (red), and meR83,152 (green). (**C**) Time evolution of RMSD of the backbone atoms of DNA fragment of DNA polymerase β with respect to the initial structure of meR83 (blue), meR152 (red), and meR83,152 (green). (**D**) Probability distribution of radius of gyration $R_{g}$ of DNA polymerase β complex: meR83 (blue), meR152 (red), and meR83,152 (green).

During the process of nucleotide incorporation, DNA polymerase β undergoes various conformational changes along its reaction pathways. The crystal structure of most of the intermediate structures was resolved experimentally. Initially, Pol β assumes an extended structure in the absence of the DNA fragment (PDBID: 1BPD) [14]. Upon DNA binding, it transforms into an open structure (PDBID: 1BPX) [15]. Subsequent binding of dNTP leads to the DNA pol β into the closed state where the N-terminal subdomain rotates and comes close to the Lyase domain (PDBID:2FMS) [35]. Here, in this study, we investigate how the methylated arginine sites affect the structural and dynamic properties of DNA polymerase β complex using the microsecond-long molecular dynamics (MD) simulation in the presence of Mg ions. The goal is to gain a deeper understanding of how methylation at specific arginine sites modulates the behavior of DNA polymerase β. Fig 1A shows the schematic representation of the simulated system, where methylated arginine sites (meR83, meR152) are shown in CPK representation. We have only looked at methylation sites that regulate DNA polymerase β’s polymerase function. Other methylated sites that affect the enzyme’s interaction with other proteins (e.g., PCNA) were not modeled.

### 3.1. Structural stability and flexibility of the methylated systems

Methylation is known to enhance DNA polymerase activity by improving DNA binding. Nevertheless, the understanding of its impact on the stability of the DNA polymerase β complex is not fully elucidated. To evaluate the structural stability and simulation convergence under methylation, we computed mean square deviations (RMSDs) of $C_{\alpha }$ atoms of protein and backbone phosphates for DNA from the initial structure. Fig 1B depicts the time evolution of RMSD of $C_{\alpha }$ atoms of protein of DNA polymerase for meR83, meR152, and meR83,152. Initial fluctuations are observed in all systems but eventually stabilize after 100 ns, indicating simulation convergence. For meR83,152, there is a sudden increase in RMSD value (4.4 Å) at 510 ns, before reaching a plateau at 550 ns. A small dip occurs at 790 ns followed by an increase, maintaining the same state up to 1000 ns. In meR152, stability is achieved at 50 ns, while meR83 displays a jump in RMSD at 400 ns (3.8 Å), gradually returns to its initial state within a few ns, and remains stable. The figure illustrates that methylation at both R83 and R152 sites enhances system flexibility, while methylation at R152 drives the protein into a more compact state compared to the WildType (WT). Mean RMSD values for meR83, meR152, and meR83,152 are 1.78 (0.29) Å, 1.75 (0.19) Å, and 2.4 (0.56) Å, respectively, while the average WT RMSD is 1.93 (0.53) Å. Fig 1C shows the time evolution of RMSD of backbone phosphate atoms of DNA fragments bound to DNA polymerase β. Similar to the protein RMSDs, the DNA phosphate backbone atoms exhibit a similar behavior. The average RMSD value of the DNA fragment for the WT system is 2.86 (0.95) Å, whereas, for the meR83, meR152, and meR83,152, the average RMSD value is 3.51 (0.76) Å, 2.98 (0.67) Å, and 5.09 (0.98) Å, respectively. A higher value was observed in the DNA fragment for all methylated systems compared to the WT. This indicates that the methylated sites interact with the backbone DNA phosphate and make it fluctuate more.

As noted above, when DNA is present, the DNA polymerase β complex demonstrates enhanced stability. A pertinent inquiry arises regarding how the methylated residues interact with various domains and subdomains of pol β in the absence of DNA. To address this question, we conducted a microsecond-long MD simulation for these systems without DNA fragments. Fig S2 (in the SI) illustrates the time evolution of RMSD of $C_{\alpha }$ atoms of DNA polymerase protein for meR83, meR152, and meR83,152. Unlike the pol β complex in the presence of DNA, the absence of DNA results in a notable jump in RMSDs. In the absence of DNA, the mean values for the methylated systems are 4.3 (0.90) Å, 10.98 (3.8) Å, and 15.01 (4.7) Å, respectively. This unequivocally indicates that in the absence of DNA, methylated residues interact differently with the protein, rendering the system less stable.

To explore the impact of methylated sites on the structural compactness of DNA polymerase β, we have calculated the radius of gyration ($R_{g}$). Fig 1D shows the probability distribution of $R_{g}$ of meR83, meR152, and meR83,152. The probability distribution of both meR83, and meR152 is sharply defined, yet the peak position differs. In contrast, the probability distribution for meR83,152 is broader. These findings suggest that DNA polymerase β undergoes a conformational transition and transits into a more closed state for meR152 as a result of methylation.

**Fig 2:**
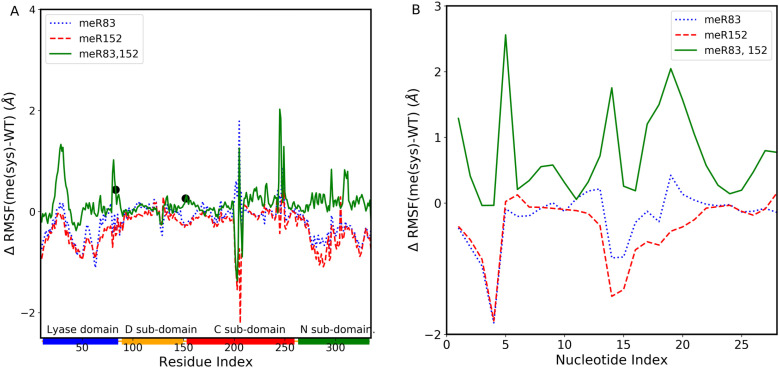
Difference in the Root Mean Square Fluctuations (RMSF) value of $C_{\alpha }$ atoms of DNA polymerase β concerning WT. (**A**) meR83 (blue dotted line), meR152 (red dashed line), and meR83,152 (green solid line). The rectangle shown in the horizontal panel reflects the DNA polymerase β domains and subdomains and are colored as follows: Lyase domain (blue) and three subdomains: D (orange), C (red), and N (green). (**B**) Difference in P atom of DNA backbone of DNA polymerase β concerning WT: meR83 (blue dotted line), meR152 (red dashed line), and meR83,152 (green solid line).

Methylation at distinct sites on the DNA polymerase β induces structural changes. To identify the subdomains most affected by the methylation, we have calculated the root mean square fluctuations (RMSF) for $C_{\alpha }$ atoms of protein residues and backbone phosphate atom of DNA. The RMSF value shows the average fluctuation of each considered atom over the total time of the simulation. Fig 2A illustrates the difference in the RMSF value of the protein for meR83 (blue dotted line), meR152 (red dashed line), and meR83,152 (green solid line) with respect to the WT, while the black circle indicates methylation sites. The figure reveals that methylation enhances RMSF values in certain subdomains while reducing them in others compared to the WT. For meR152, RMSF values of subdomains decreased, whereas methylating R83 significantly enhances residues that correspond to the catalytic subdomain. In both meR83 and meR152, the RMSF values for the Lyase domain, DNA binding subdomain, and N-terminal base pairing subdomain decrease, indicating a transition of DNA pol β to a more compact state. Simultaneous methylation at both R83 and R152 increases the RMSF value of the residues linking the Lyase domain and DNA binding subdomain, as well as the linker region between the DNA binding subdomain and catalytic subdomain. Additionally, the RMSF value of the residues corresponds to the catalytic subdomain, and the N-terminal base pairing domain also increased. On the contrary, in the absence of DNA, notable changes occur due to methylation. Fig S3 (in SI) displays the RMSF value of the protein for meR83, meR152, meR83,152, and WT in the absence of the DNA. In the absence of DNA, methylating both sites increases the RMSF value almost 3-fold compared to in the presence of DNA. We also looked at the impact of methylation on the DNA fragment. Fig 2B demonstrates the difference in RMSF value of the DNA fragment for meR83, meR152, meR83,152 from WT. While methylation at the individual sites has a modest impact on DNA fragment RMSF, simultaneous methylation at both R83 and R152 results in a significant increase in fluctuations, possibly attributed to methyl group interactions with the DNA backbone atoms.

The crystal structure of DNA polymerase β revealed that the enzyme adopts a closed structure facilitated by a hydrogen bond (H-bond) between S44 (located in the Lyase domain) and E335 (lies in the N-terminal base paring subdomain). This H-bond is absent in experimentally resolved structures where DNA was absent. This observation implies that both DNA and this H-bond interaction play a vital role in stabilizing the enzyme in the closed state. To analyze how methylation influences this H-bond, we looked at the H-bond occupancy using our MD trajectory. The H-bond occupancy is defined as the fraction of time during which the residue S44 forms a H-bond with the S335 residue. The GROMACS hbond tool was employed to compute the H-bond occupancy with a threshold distance of 3.5 Å and an angle cutoff of 30^0^. Results indicate that the H-bond was present in the WT system for 80% of the simulation duration, while methylation does impact the H-bond occupancy. Specifically, the H-bond occupancy for the methylated systems is 75% (meR83), 55% (meR152), and 40% (meR83, 152), respectively. The average donor-acceptor distance between S44 and E335 for all methylated systems compared to WT is presented in Table S1 (in SI). The reduction in H-bond occupancy for the methylated system compared to WT is likely associated with the presence of the methyl group.

Furthermore, an important question arises: does methylation impact the polar interactions between different domains and subdomains? To address this, we calculated the number of H-bonds formed between the methylated systems and the various domains of DNA pol β complex. We found the number of H-bonds varied throughout all three simulated systems. Specifically, for meR83, the Lyase domain formed an average of 2.3 (0.7) H-bonds with the methylated residues, while the D-subdomains formed 0.2 (0.006) H-bonds, and no H-bonds were observed with other subdomains. For meR152, an average of 1.3 (0.4) H-bonds were formed with the C-subdomain, and no H-bonds were detected between different subdomains of the enzyme. In the case of meR83,152, the Lyase domain formed an average of 1.9 (0.9) H-bonds, the D-subdomain formed 0.01 (0.002) H-bonds, and the C-subdomain formed 1.6 (0.6) H-bonds. Interestingly, when both R83 and R152 were methylated, the average number of H-bonds meR83,152 formed with the Lyase domain decreased, while the H-bonds formed between meR83,152 and the C-subdomain increased. Additionally, we explored how methylation affects the DNA fragment by calculating the center of mass (COM) distances between methylated systems (meR83, meR152, and meR83,152) and DNA. The findings revealed that DNA comes closer to the methylated residues compared to the WT. The average COM distance between meR83, meR152, and meR83,152 are 32.6 Å (1.1), 34.7 Å (1.1), and 26.3 Å (1.4), respectively, while, for WT is 34.9 Å, 35.8 Å, and 36.3 Å.

Experimental evidence suggests that methylation affects the DNA binding property of the DNA polymerase β complex. This raises an additional question: does methylation impact the interaction between DNA and different subdomains of the enzyme? To address this, we computed the number of H-bonds formed between DNA and various subdomains. For meR83, meR152, and meR83,152, the average number of H-bonds between DNA and D-subdomain is 2.9, 3.0, and 3.1; between DNA and C-subdomain is 4.6, 4.9, and 5.9; and between DNA and N-subdomain is 4.2, 4.8, and 5.2. In contrast, for WT, the average number of H-bonds between DNA and D-subdomain is 2.7; DNA and C-subdomain is 4.6; DNA and N-subdomain is 2.7. These findings suggest that methylation causes DNA to move closer to the polymerase domain due to a significant increase in the number of H-bonds compared to WT. The time evolution of H-bonds between DNA and different subdomains for all three methylated systems is depicted in Fig S4 (in SI). These results underscore the crucial role of H-bonds in the structural transitions observed in all three methylated systems, which will be discussed in the following section.

## 4. Principal Component Analysis

The principal component analysis (PCA) results in terms of eigenvalues (3N = 3  ×  354 = 1062, 326 $C_{\alpha }$ and 28 P atoms for DNA fragment) and eigenvector obtained by diagonalizing the covariance matrix of atomic fluctuations for all the three methylated arginine systems are shown in Fig S5 (in SI). The corresponding eigenvalues of each eigenvector are plotted in decreasing order. Upon comparing the three systems, it is observed that the first few principal components (PCs) describing the properties of motion were not identical. In meR83,152, the magnitude of the first few eigenvalues was higher compared to meR83 and meR152. The first few 20 eigenvectors describe the collective motion of methylated DNA pol β complex and account for approximately ~ 80%, 74%, and 90% of the overall motion in meR83, meR152, and meR83,152, respectively, whereas for WT it is 82%. In meR83,152, the first few PCs describing cooperative motions are larger than in meR83, and meR152, as well as WT. The first two eigenvectors account for approximately ~39.8%, 29.7%, and 59.2% of the total collective motion of meR83, meR152 and meR83,152, respectively. Moreover, Supplementary Fig S6 (in SI) shows the residue-wise contribution of PC1, and PC2 for meR83, meR152 and meR83,152. In contrast, in the absence of DNA, these value increases for all three simulated systems meR83 (~58%), meR152 (~85%), and meR83,152 (~90%), respectively. The increased overall collective motion of meR152 and meR83,152 in the absence of DNA, particularly in the first two PCs, may be attributed to the presence of R152 in the linker region between the D-Subdomain and C-subdomain, where DNA binds. The interaction with meR152 stabilizes the system in the presence of DNA. Overall, methylating both R83, and R152 enhances the collective motions of DNA polymerase β, aligning with the cross-correlation results (see next section).

**Fig 3:**
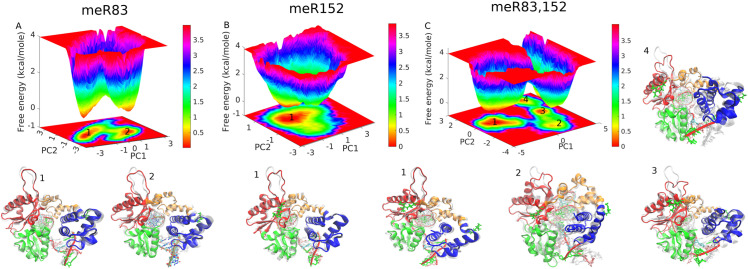
Free energy landscape of DNA polymerase β projected along the principal component 1 (PC1) and principal component 2 (PC2) (**A**) meR83, (**B**) meR152, and (**C**) meR83,152. Representative structures corresponding to the minimum energy basin are also shown. The minimum energy basins for all three studied systems are highlighted using the number.

To investigate the impact of methylation on the DNA pol β complex, we utilized the first two PCs obtained from the PCA analysis to generate the free energy landscape (FEL) for all three methylated systems, as depicted in Fig 3. The FEL patterns exhibit distinct basin configurations for each of the three methylated systems, as is evident from Fig 3. In the case of meR83,152, the conformational space displays multiple minimum free energy basins with a broad structural distribution. Conversely, meR152 showed a single broad and stable global minimum confined within a particular basin on the free energy surface. The meR83 system reveals two distinct minimum free energy states. These findings indicate that in the three complexes, meR83,152 attains more conformations compared to meR83, and meR152. The minimum free energy basin observed in FEL is labeled using the numbers. The structures corresponding to these basins are shown in snapshots and contrasted with the WT state of the enzyme.

### 4.1. New salt-bridge formed due to the methylation

PCA and H-bonds analysis reveals that methylated residues induced structural transitions in DNA pol β complex. Consequently, methylated residues are expected to form new salt bridges with neighboring residues and different subdomains. We conducted a salt bridge analysis in the presence and absence of DNA fragments. Table 1 shows the list of salt bridges formed between the methylated residues and the different domains/subdomains of DNA polymerase β.

**Table 1:** List of the salt bridges formed between the methylated residues and the different domains/subdomains of DNA polymerase β. The percentage of time each salt bridge was observed during the simulation is shown in brackets.

System	Salt Bridges
**meR83 with DNA**	meR83-E71 (1.2), meR83-E75 (2.5), meR83-E86 (73.04)
**meR152 with DNA**	meR152-E153 (0.54), meR152-E186 (2.83)
**meR83,152 with DNA**	meR83-E75 (0.79), meR83-E86 (60.78), meR152-E153 (0.61), meR152-E186 (3.72)
**meR83 without DNA**	meR83-E75 (6.12), meR83-E86 (78.63)
**meR152 without DNA**	meR152-E153 (2.23), meR152-E186 (3.98)
**meR83,152 without DNA**	meR83-D116 (0.2), meR83-E86 (52.63), meR83-E75 (0.72), meR83-D92 (0.46), meR83-E335 (0.01), meR152-E153 (0.72), meR152-E186 (15.24)

In the presence of DNA, meR83 forms a new salt bridge with E71, which was not present in the absence of DNA, indicating that the presence of DNA stabilizes the Lyase domain. Compared to meR83, when both residues are methylated, the salt bridge formed with E71 is broken. This occurs because meR152 induces a twist in the helical region of the Lyase domain, causing the salt bridge between meR83 and E71 to break. This could indicate that methylation may occur sequentially with meR83 occurring first to stabilize the lyase domain, followed by meR152 to induce salt-bridge breakage to relieve this stabilization. In meR83,152, with DNA, the methylated residues form four salt bridges with residues located in the Lyase domain or C-subdomain, whereas, without DNA, it forms seven salt bridges. Notably, salt bridges formed with residues located in the D- and N-subdomains are absent in the presence of DNA, likely due to the DNA fragments interacting with these subdomains and increasing the distance from the methylated residues. Fig S7 (in SI) illustrates the schematic representation of salt bridges formed in both the presence and absence of DNA for the meR83,152 system. To investigate the effect of methylation on salt bridge formation, we calculated the average O-N distance between the methylated arginine and E75, E86, E153, and E186 residues in the presence of the DNA and compared with the WT. We observed that methylating residues decrease the distance between these residues. In the meR83,152 system, the average distance between meR83 and E75 is 9.4 Å, between meR83 and E86 is 5.9 Å, between meR152 and E153 is 8.1 Å, and between meR152 and E186 is 7.3 Å, while for the WT, the average distance between meR83 and E75 is 7.8 Å, between meR83 and E86 is 7.5 Å, between meR152 and E153 is 9.3 Å, and between meR152 and E186 is 8.0 Å, respectively. These results suggest that in the methylated system, the Lyase domain expands while the D-subdomain and C-subdomain transition to a more compact state due to the interaction with DNA.

To explore how the methylated residues influence the polymerase domain, we examined the interaction of the methylated system with active sites (D190, D192, D256) with surrounding residues. Our analysis revealed that methylation induces the formation of a new salt bridge between D190 and R254 which is absent in the WT system. This interaction leads to the C-subdomain approaching the D-subdomain, resulting in a more rigid polymerase domain compared to the WT.

### 4.2. Cross-correlation map shows the correlation enhanced between different subdomains upon methylating both R83 and R152

To characterize how the methylation affected the coupling between the distant subdomain of the DNA pol β complex, we calculated the cross-correlation between the positions of different residues in 3-dimensional space. This method is often used to study the allosteric effect on ligand binding [51–54]. Conventional implementations (Pearson Correlation Coefficient) compute the dynamic cross-correlation map (DCCM) of the position vector $C_{\alpha }$ atoms of amino acids [46]. However, DCCM ignores correlated motion in orthogonal directions [55]. This problem can be avoided by using linear mutual information (LMI) based cross-correlation metric, which we use in this study. The cross-correlation matrix elements are, $C_{ij}\left(r_{i},r_{j}\right)$ is defined as


$$C_{ij}\left(r_{i},r_{j}\right)={\left(1-e^{-\left(2/d\right)I_{ij}}\right)}^{-1/2},$$ (2)

where *d* is equal to 3. The coefficient of $C_{ij}$ is zero for fully uncorrelated motions and assumes values up to 1 for fully correlated motions. The $I_{ij}$ is the LMI calculated as


$$I_{ij}=H_{i}+H_{j}-H_{ij},$$ (3)

where H is closely related to the Shannon entropy,


$$H_{i}=-\int p\left(r_{i}\right)\ln p\left(r_{i}\right)dr_{i}$$

$$H_{ij}=-\iint p\left(r_{i},r_{j}\right)\ln p\left(r_{i},r_{j}\right)d\left(r_{i}\right)d\left(r_{j}\right)$$ (4)

where, *i* and *j* corresponds to residue *i* and residue *j*, $r_{i}$ and $r_{j}$ are the 3D Cartesian vector of atomic coordinates of the corresponding $C_{\alpha }$ for proteins, *P* atoms for DNA fragments, whereas $p(r_{i)}$ and $p(r_{i},r_{j})$ shows, respectively, the marginal probability density for $r_{i}$ and the joint probability density of $r_{i}$ and $r_{j}$ [51,56]. The PCA analysis reveals that conformational transitions in DNA pol β are influenced by methylation. Comparing the LMI cross-correlation among subdomains aids in identifying residues exhibiting distinct behavior across various DNA pol β conformations. Notably, the difference in correlation between the WT state and methylated states can pinpoint the residues gaining or losing contact due to the methylations, consequently initiating the conformational transitions. In our subsequent analysis, we incorporated an unbiased trajectory, comparing the correlation heat maps of all residues in the WT form with the three methylated systems (meR83, meR152, and meR83,152), to understand the coupling between the subdomains and protein residue fluctuations as a consequence of methylation.

**Fig 4:**
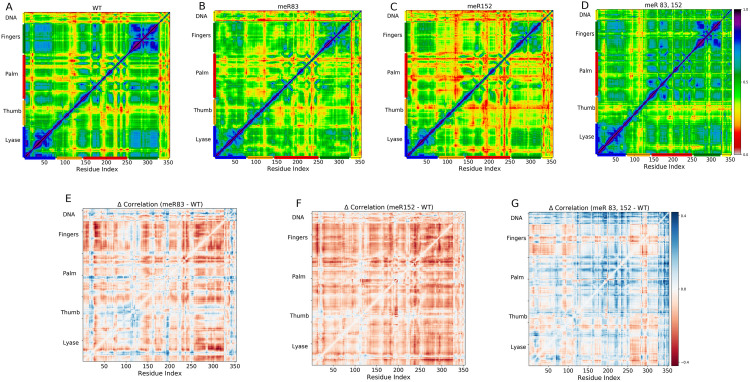
(A-D) Cross correlation matrices of the fluctuations of the $C_{\alpha }$ atoms and phosphates atoms of DNA polymerase β for four systems (A) WT; (**B**) meR83; (**C**) meR152; (**D**) meR83,152) computed using LMI. (**E-G**) The difference in cross-correlation matrix element for the (**E**) meR83, (**F**) meR152, and (**G**) meR83,152 with respect to the WT. Highly correlated blocks (G) indicate that methylating both residues shows a signature of long-distance correlation motions.

Fig 4 illustrates the cross-correlation map for WT **(A)**, meR83 **(B)**, meR152 **(C)**, and meR83,152 **(D)**. The LMI correlation is notably higher for meR83,152 compared to WT, as indicated by the increased prevalence of blue and green colors (Fig 4A and 4D). Both meR83 and meR152 exhibit weak intra-subdomain correlation, with meR152 displaying almost no correlations between the D-subdomain and N-subdomain. In contrast, methylating both R83, and R152 enhances both inter- and intra-subdomain correlations. Notably, a substantial increase in LMI values between the protein domains and DNA fragments upon methylation is observed, consistent with the experimental findings that methylation enhances DNA binding.

Fig 4(E-G) displays the change in correlation coefficient (*Δ* correlation) relative to the WT for the three methylated systems. Residues gaining correlations are depicted in blue color while those losing correlations are shown in red color. It is evident from the figure methylating R152 site results in residues corresponding to different domains and sub-domains losing correlations, whereas meR83 shows inter-subdomain loss of correlation between various subdomains, especially Lyase domain and N-subdomains and C- and N-subdomain. Methylating both R83 and R152 demonstrates a gain in correlation between different domains and subdomains. Intriguingly, methylation increases the correlation between the protein and DNA fragment, except for the meR152 system, possibly due to the location of R152. Additionally, in the case of meR83,152, a significant gain in correlation is observed in the linking region of the C- and N-subdomain, which is quite far from the methylated sites. This clearly shows the methylation-induced long-range correlation in the DNA pol β complex.

### 4.3. Structural Network Analysis

**Fig 5:**
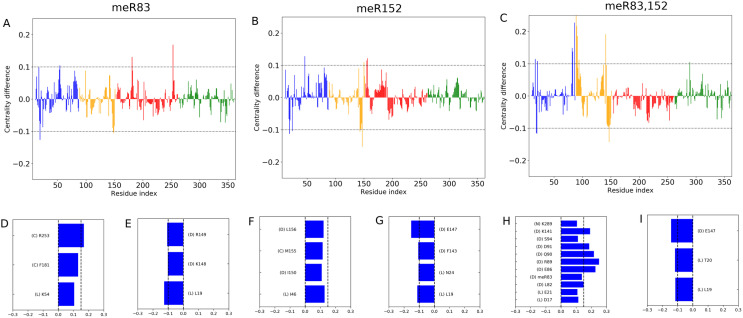
(A-C) The values of the difference in betweenness centrality (BC) with respect to the WT state plotted as a function of residue index for three systems(A), meR83; (B), meR152; (C), meR83,152. The horizontal lines correspond to the value ±0.1. The data are colored according to domain/subdomain color as shown in Fig 1A. Data shows a large change in normalized BC value due to the methylation. Residues with the largest positive change in normalized BC value for (**D**) meR83, (**F**) meR152, and (**H**) meR83,152. Residues with the largest negative change in normalized BC value for (**E**) meR83, (**G**) meR152, and (**I**) meR83,152. The DNA polymerase β subdomains corresponding to those residues are also mentioned next to the residue number.

As seen above, methylation induced structural transition in the DNA polymerase β complex. To gain more profound insight related to the functional movement and information exchange mechanism, we built a protein connectivity graph network model (GNM). This methodology involves a weighted network whose nodes represent the $C_{\alpha }$ atoms of the amino acids in the protein and phosphate atom in DNA, and the correlation between them is the edges connecting the nodes. We then calculated the betweenness centrality (BC), a graph theoretical measure that provides a way to quantify the amount of information that flows via the nodes and edges of a network. If a node *i* acts as a bridge between two other nodes along the shortest path joining them, then the BC of a given node *i* is given by the following equation:


$$BC\left(i\right)=\underset{ab}{\sum }\frac{n_{ab}^{i}}{g_{ab}},$$ (5)

where $g_{ab}$ represents the total number of shortest paths joining nodes *a* to *b*, out of which $n_{ab}^{i}$ path pass through node *i* [52]. By analyzing the BC values, we can identify the residues that are involved in the information-propagating pathway. Centrality analysis has been extensively employed to determine the functionally significant residues in communication pathways, metabolic networks, disease networks, and allosteric communications [57–60]. The change in BC has been used to explore the dynamics of the spike protein [61]. In this work, we used the difference in BC as a metric to identify key residues that gain or lose relative importance along the information pathway due to methylation. The difference in the normalized BC is measured by comparing the methylated states with the WT (i.e., BC^*meR*^ - BC^*WT*^, for every residue and nucleotide of DNA polymerase β complex) from our all-atom trajectory with explicit solvation. Fig 5(A-C), displays the difference in BC value per residues for the methylated system in comparison to the WT. The result indicates that some residue loss and others gain importance along the information pathways.

For meR83, and meR152 the residues with significant (e.g., >0.1) change in the BC value are mostly from the D-subdomain or C-subdomain, whereas, for meR83,152 the significant change in BC value is either from the D-subdomain or the N-subdomain (Fig 5D-I). This suggests that allosteric information flows through Lyase and the C-subdomain for meR83, whereas for meR152, and meR83,152, the information flows through the Lyase and the D-subdomain. Any mutation in these regions can break the allosteric network and affect the functionality of DNA pol *β* complex. This further supports that the methylation may occur sequentially with meR83 occurring first to stabilize the lyase domain and hasten DNA repair, followed by meR152 to induce salt-bridge breakage and relieve this stabilization.

In culmination, the most interesting aspect is the striking large change in BC of the residues that are distant from the methylated sites in the 3D structure. Significant gain or loss of BC is observed in residues R253, R149, and L19, whereas, for meR83.152, it is D17, L19, T20, E21, K249. There is no significant loss or gain is observed in the meR152 system. For meR83, 152, the first 5 residues correspond to the Lyase domains whereas K249 corresponds to the loop region of the catalytic subdomain. For the meR83 system, the residue R253 corresponds to the *β* -sheet region of the catalytic subdomain, and R149 corresponds to the linker region of the DNA binding subdomain and Lyase domain. These results suggest that methylation has a long-range effect and not just on the methylation target site in the Lyase domain, the effect of this perturbation felt by the residues corresponds to the catalytic subdomain.

### 4.4. Methylation R83, R152, and R83,152 together changes the effector/sensitivity profile of DNA polymerase β complex

**Fig 6:**
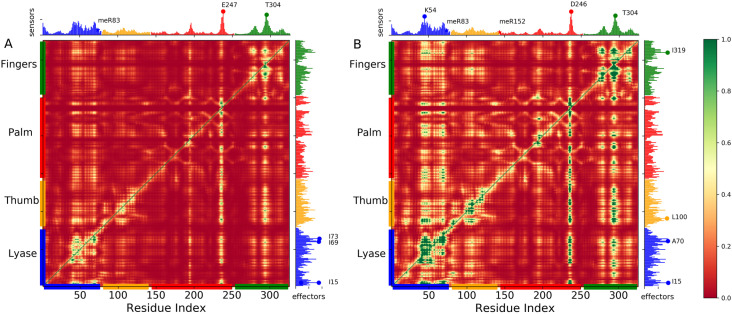
Perturbation response analysis identifies the highly influential and sensitive residues that likely propagate allosteric signals due to the methylation in meR83 and meR83,152. PRS maps ((**A**) meR83 and (**B**) meR83,152) indicate the strongest perturbation response sites as shown by the color map (see the scale on right). The peaks in the curves along the axes indicate the effectors (left ordinate) and sensors (upper abscissa). DNA polymerase β subdomains are color-coded along the lower abscissa highlighting lyase (blue), thumb (orange), palm (red), and finger subdomains (green), as mentioned in Fig 1A.

Centrality network reveals that methylation modulates the information pathways of DNA polymerase β complex. To investigate the impact of methylation on the residues responsible for the long-range transmission signal, we used the perturbation response scanning (PRS) method on the meR83, and meR83,152 systems. Anisotropic network model (ANM) from the representative structures (every 10 ns interval) of meR83, and meR83,152 was constructed, representing the protein as elastic mass and springs networks. The PRS map was generated using the covariance matrices from the first 20 nonzero ANM frequency modes. These lower frequency modes reveal the collective changes in structure close to the native state. The PRS method (details are given in the method section) probes the response of each residue to perturbation in every other residue. Fig 6 displays the PRS maps of meR83 (**A**) and meR83,152 (**B**), drawn using the MD trajectory. The representative structures were taken over every 10 ns interval resulting in a total of 100 representative structures. The present map is the averaged PRS map generated using 100 representative structures. Each element in the PRS matrix represents the response of residue *j* to a perturbation on residue *i*. The rows of the PRS map provide information about the influence of a particular residue in transmitting signals when subjected to the unit perturbation, while the column provides information about the sensitivity of a given residue to those signals. The effector residues (peaks in the right ordinate bar plot) and sensor residues (peaks in the upper abscissa bar plot) are colored by domain/subdomain identity in the panel.

The results show that the perturbation response of the DNA pol β remains relatively unchanged due to the methylation (meR83, and meR83,152), but specific regions show significant change upon methylation. Both effector and sensor residues signal enhanced compared to WT (Fig S8 in SI). In the case of meR83, there is a strong enhancement in sensitivity for E247 (C-subdomain) and T304 (N-subdomain), but the signal in other domains is relatively weak. However, methylating both R83 and R152, the sensitivity of E247 is suppressed and a new signal emerges in the Lyase domains (see Fig 6B). Upon methylation, the most affected sensors are clustered in the N-subdomain and Lyase domain, while for WT, the most affected sensors clustered in the C-subdomain. The effectors are clustered in the Lyase domain for meR83, while methylating both a few new signals emerge in the D-subdomain (L100) and N-subdomain (I319). Our results demonstrate that the methylation has a long-range effect, R83 lies on the Lyase domain and R152 likes in the D-subdomain but the strong effector and sensor signal emerges in the C-subdomain and N-subdomain compared to the WT.

## 5. Conclusion

We performed MD simulations to investigate the effect of methylation on the structural dynamics of DNA polymerase β both in the presence and absence of DNA. Our results reveal that the methylating R83 and R152 in DNA polymerase β has led to significant structural changes that could potentially enhance the polymerase activity. The impact of methylation on the protein differs considerably depending on whether DNA is present or not. In the presence of DNA, the DNA polymerase β becomes more stable whereas in its absence, the protein undergoes significant fluctuations, with RMSF value experiencing an approximately threefold increase compared to in presence of DNA. PCA analysis reveals that methylating both the sites, multiple minimum free energy basins populated while the meR152 system adopts more compact states. H-bonds analysis demonstrates that the DNA moves closer to the C- and N-subdomain indicating that the system is moving more compact state. The formation of a new salt bridge between D190 and R254 suggests that methylation induces proximity between the C-subdomain and N-subdomain.

To understand the long-distance coupled motions between different subdomains of DNA polymerase β, a cross-correlation metric based on LMI was employed. Methylation was found to enhance both inter- and intra-subdomain correlation in DNA polymerase β with a substantial increase in LMI value observed between the protein domains and DNA fragments, indicating a strong binding affinity. Moreover, methylation induces long-range correlations within DNA polymerase β. The analysis of the graph connectivity networks reveals that methylation modulates the information pathway and identifies the residues exhibiting long-distance coupling with the methylated sites. In summary, our results offer an atomic-level comprehension of the structural transitions induced by methylation, shedding light on the mechanisms behind methylation-induced enhancement of activity in DNA polymerase β. Although MD simulations provide detailed insight, they are inherently limited by simulation timescales and cannot fully replicate complex in vivo environments. One major challenge is the limited availability of experimental data that directly links specific methylation sites to structural transitions. Experimental techniques like X-ray crystallography and NMR spectroscopy often struggle to capture transient, dynamic structural changes associated with PTMs. Future work incorporating such as enhanced sampling techniques and multiscale modeling approaches alongside validation could provide a more comprehensive understanding of how methylation drives these structural transitions.

## Supporting information

S1 Fig Flow chart.
Flow chart of MD Simulation.
(DOCX)

S2 Fig Time evolution of Root Mean Square Deviation (RMSD).
Time evolution of RMSD of Cα atoms of DNA polymerase β in the absence of DNA with respect to the initial structure of meR83 (blue color), meR152 (red color), and meR83, 152 (green color).
(DOCX)

S3 Fig Root Mean Squared Fluctuations (RMSF).
The RMSF of meR83 (blue color), meR152 (red color), meR83,152 (green color), and WT (black color) in the absence of DNA. The rectangles shown in the horizontal panel reflect the DNA polymerase major sub-domains, which are colored as follows: Lyase domain (blue), and three sub-domains: D (orange), C (red), and N (green).
(DOCX)

S4 Fig Time evolution of hydrogen bonds.
Time evolution of Hydrogen bonds (A) DNA and DNA binding subdomain, (B) DNA and Catalytic subdomain, and (C) DNA and N-base pairing subdomain. The blue and red solid line shows the hydrogen bond for meR83 and meR152, whereas the green line shows the hydrogen bond for meR83,152.
(DOCX)

S5 Fig Scree plot.
Scree plot for principal component analysis on the MD data of DNA pol β complex (A) meR83, (B) meR152, and (C) meR83,152.
(DOCX)

S6 Fig Each residue contribution to Principal Component 1 (PC1) and principal component 2.
DNA polymerase β each residue contribution to PC1 and PC2 of (A) meR83, (B)meR152, and (C) me83,152. The data are colored according to domain/subdomain color as shown in Fig 1A (in the main text).
(DOCX)

S7 Fig Schematic representation salt-bridges.
Schematic representation of salt -bridges formed between the methylated residues and different domains and sub-domains shown on the DNA pol β structure (A) meR83,152 without DNA, and (B) meR83, 152 with DNA. Residues form salt bridges, as shown in stick representations.
(DOCX)

S8 Fig Perturbation response analysis.
Perturbation response analysis identifies the highly influential and sensitive residues that likely propagate allosteric signals in all three methylated systems. The effector provides information about the influence or effectiveness of a particular residue in transmitting signals when subjected to unit perturbation, while the sensor provides information about the sensitivity of a given residue to those signals. The effector signal of (A) WT and (B) meR83,152. The sensor signal of (C) WT and (D) meR83, 152.
(DOCX)

S1 Table Donor-acceptor distance.
The donor-acceptor distance between S44 and E335 for all systems.
(DOCX)
