# Supplementary figures and images for: Deciphering the structural consequences of R83 and R152 methylation on DNA polymerase β using molecular modeling
Source: PLoS One. 2025 Mar 12;20(3):e0318614. doi: 10.1371/journal.pone.0318614 (PMC11902276; doi:10.1371/journal.pone.0318614)

**S1 Fig.**

**
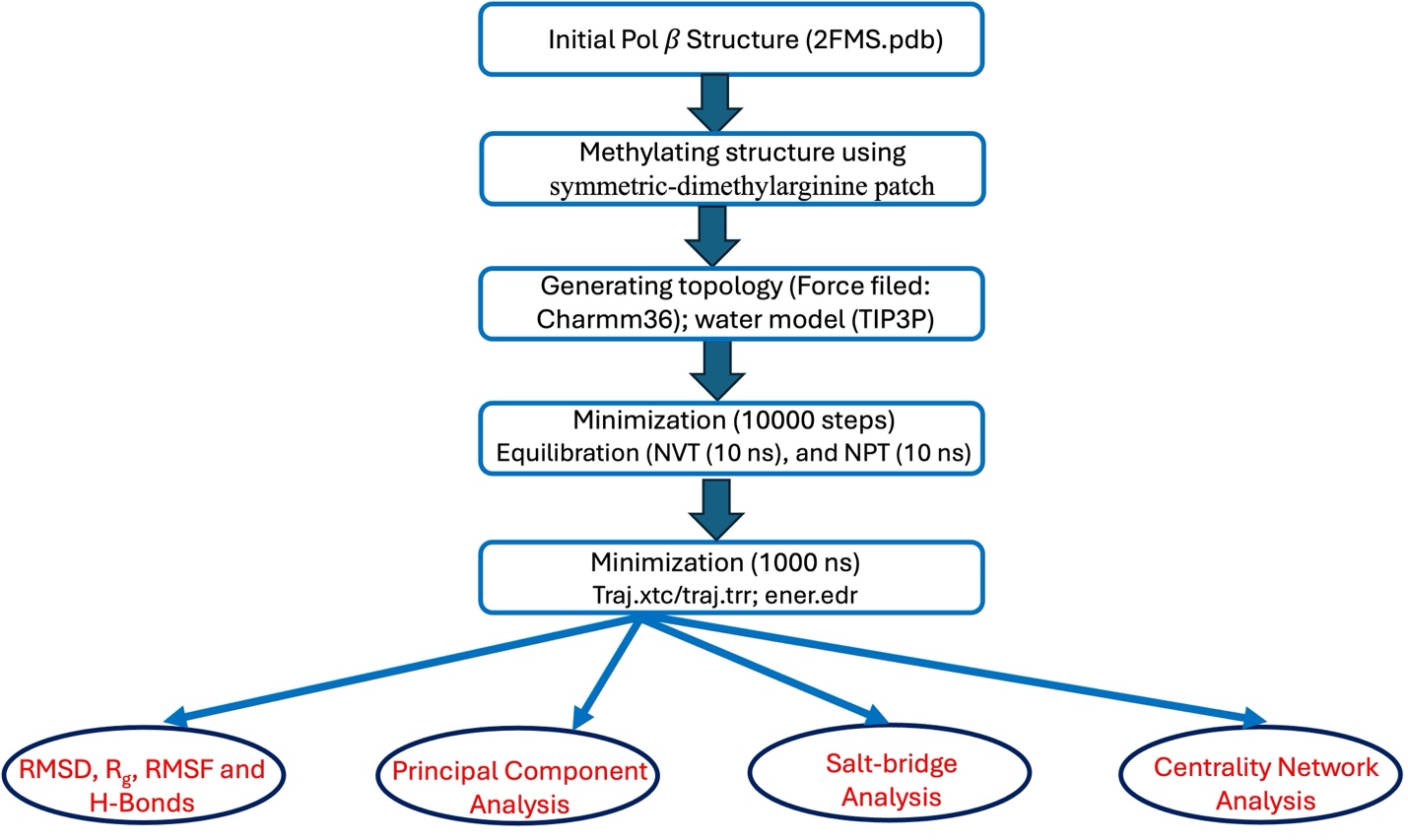
**

**Flow Chart.** Flow chart of MD Simulation.

Supplement: S1 Fig — Flow chart of MD Simulation. (DOCX) [file pone.0318614.s001.docx]
