# Supplementary material for: Deciphering the structural consequences of R83 and R152 methylation on DNA polymerase β using molecular modeling
Source: PLoS One. 2025 Mar 12;20(3):e0318614. doi: 10.1371/journal.pone.0318614 (PMC11902276; doi:10.1371/journal.pone.0318614)
Supplement: S2 Fig — Time evolution of RMSD of Cα atoms of DNA polymerase β in the absence of DNA with respect to the initial structure of meR83 (blue color), meR152 (red color), and meR83, 152 (green color). (DOCX) [file pone.0318614.s002.docx]

**S2 Fig.**

**
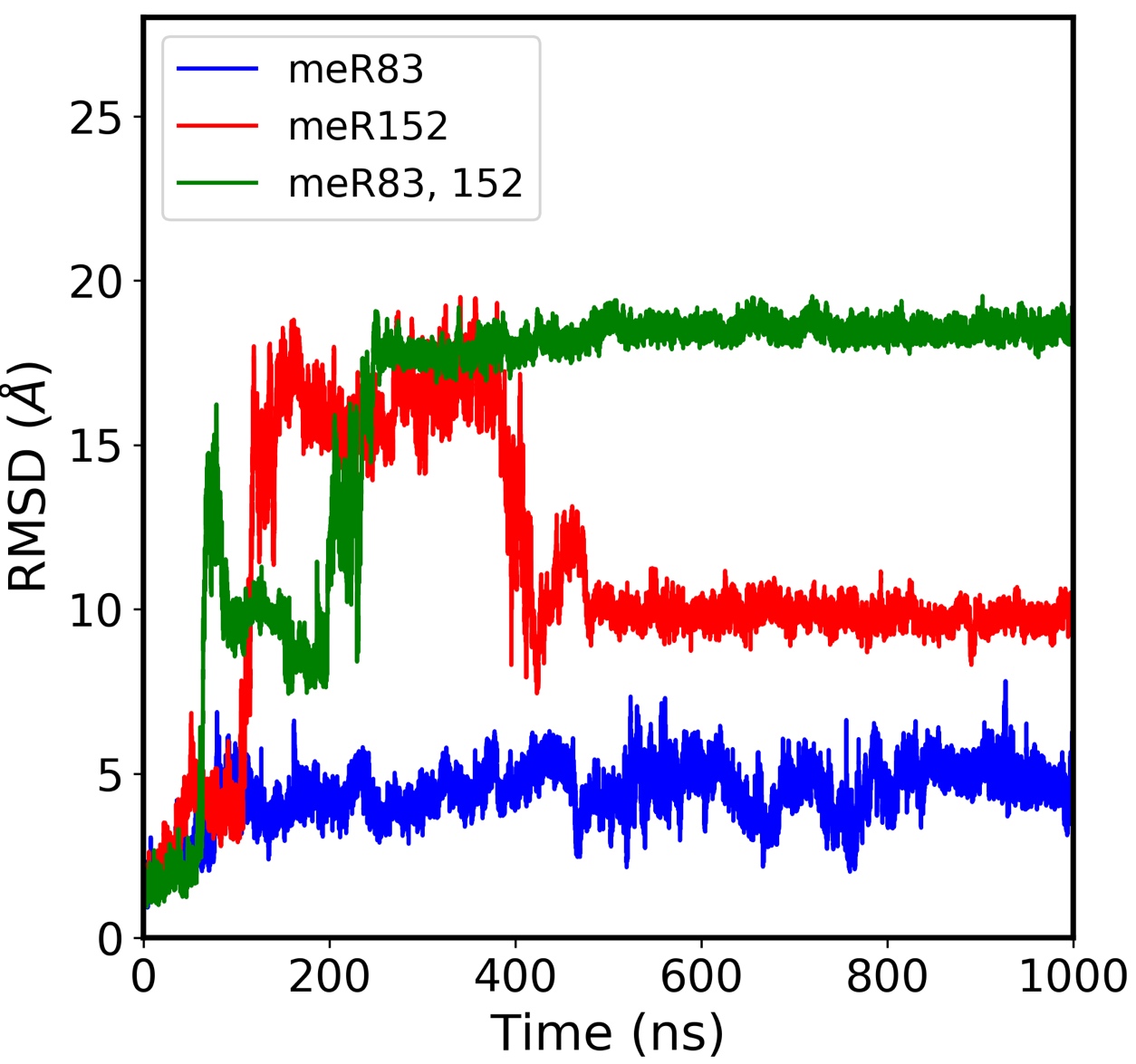
**

**Time evolution of Root Mean Square Deviation (RMSD).** Time evolution of RMSD of Cα atoms of DNA polymerase β in the absence of DNA with respect to the initial structure of meR83 (blue color), meR152 (red color), and meR83, 152 (green color).
