# Supplementary material for: Deciphering the structural consequences of R83 and R152 methylation on DNA polymerase β using molecular modeling
Source: PLoS One. 2025 Mar 12;20(3):e0318614. doi: 10.1371/journal.pone.0318614 (PMC11902276; doi:10.1371/journal.pone.0318614)
Supplement: S3 Fig — The RMSF of meR83 (blue color), meR152 (red color), meR83,152 (green color), and WT (black color) in the absence of DNA. The rectangles shown in the horizontal panel reflect the DNA polymerase major sub-domains, which are colored as follows: Lyase domain (blue), and three sub-domains: D (orange), C (red), and N (green). (DOCX) [file pone.0318614.s003.docx]

**S3 Fig.**

**
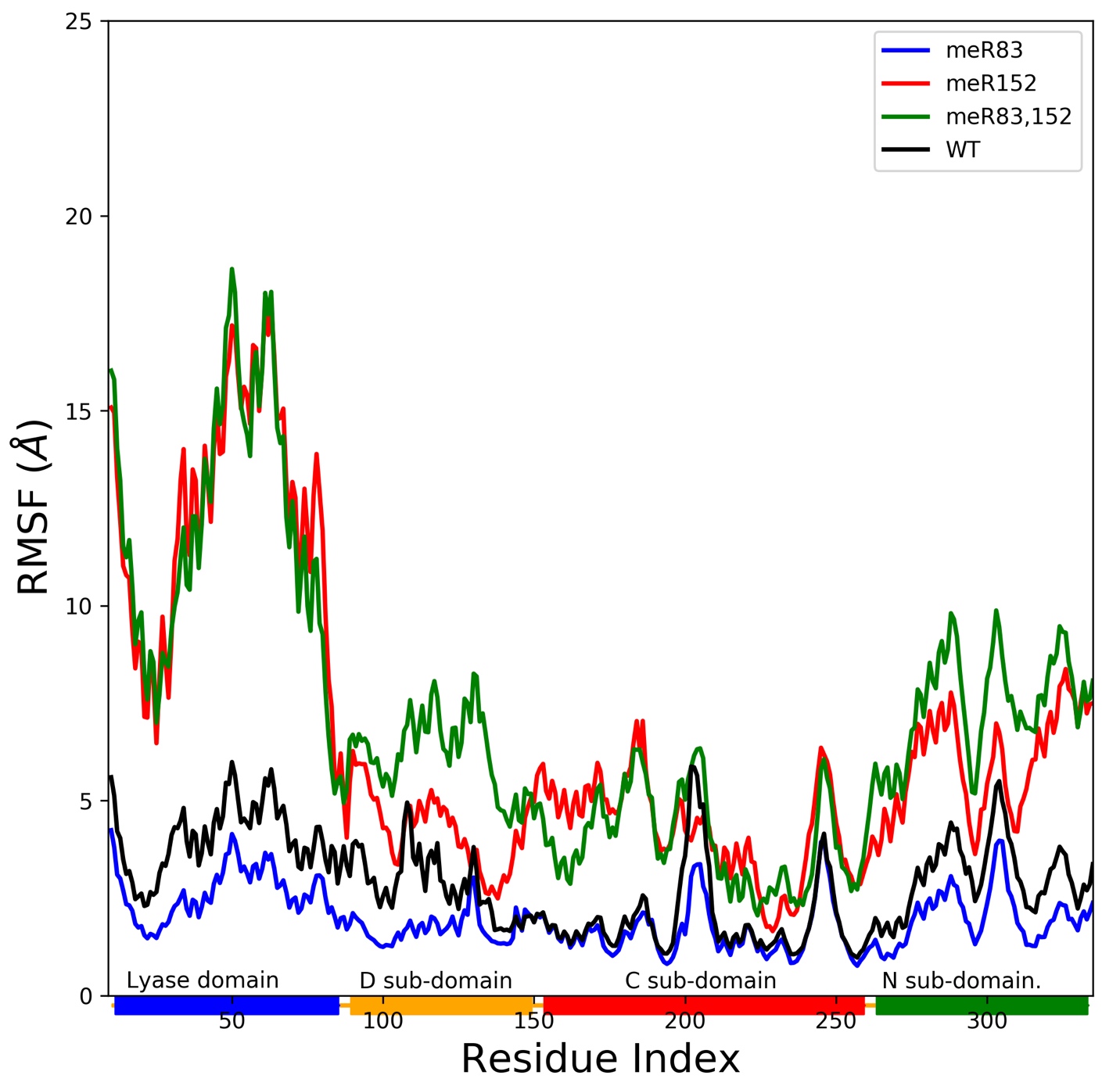
**

**Root Mean Squared Fluctuations (RMSF).** The RMSF of meR83 (blue color), meR152 (red color), meR83,152 (green color), and WT (black color) in the absence of DNA. The rectangles shown in the horizontal panel reflect the DNA polymerase major sub-domains, which are colored as follows: Lyase domain (blue), and three sub-domains: D (orange), C (red), and N (green).
