# Supplementary material for: Deciphering the structural consequences of R83 and R152 methylation on DNA polymerase β using molecular modeling
Source: PLoS One. 2025 Mar 12;20(3):e0318614. doi: 10.1371/journal.pone.0318614 (PMC11902276; doi:10.1371/journal.pone.0318614)
Supplement: S4 Fig — Time evolution of Hydrogen bonds (A) DNA and DNA binding subdomain, (B) DNA and Catalytic subdomain, and (C) DNA and N-base pairing subdomain. The blue and red solid line shows the hydrogen bond for meR83 and meR152, whereas the green line shows the hydrogen bond for meR83,152. (DOCX) [file pone.0318614.s004.docx]

**S4 Fig.**

**
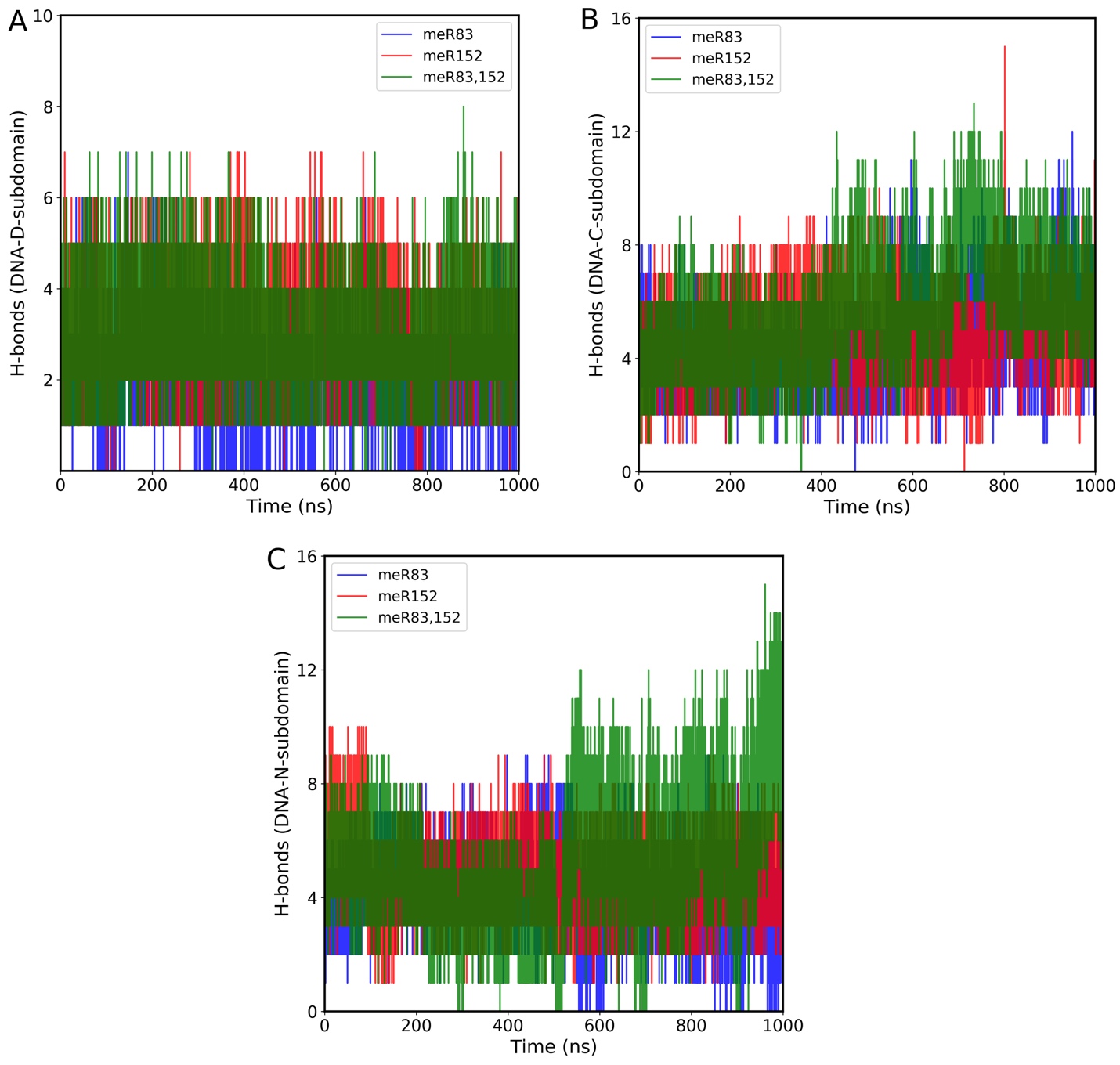
**

**Time evolution of Hydrogen Bonds.** Time evolution of Hydrogen bonds (A) DNA and DNA binding subdomain, (B) DNA and Catalytic subdomain, and (C) DNA and N-base pairing subdomain. The blue and red solid line shows the hydrogen bond for meR83 and meR152, whereas the green line shows the hydrogen bond for meR83,152.
