# Supplementary material for: Deciphering the structural consequences of R83 and R152 methylation on DNA polymerase β using molecular modeling
Source: PLoS One. 2025 Mar 12;20(3):e0318614. doi: 10.1371/journal.pone.0318614 (PMC11902276; doi:10.1371/journal.pone.0318614)
Supplement: S5 Fig — Scree plot for principal component analysis on the MD data of DNA pol β complex (A) meR83, (B) meR152, and (C) meR83,152. (DOCX) [file pone.0318614.s005.docx]

**S5 Fig.**

**
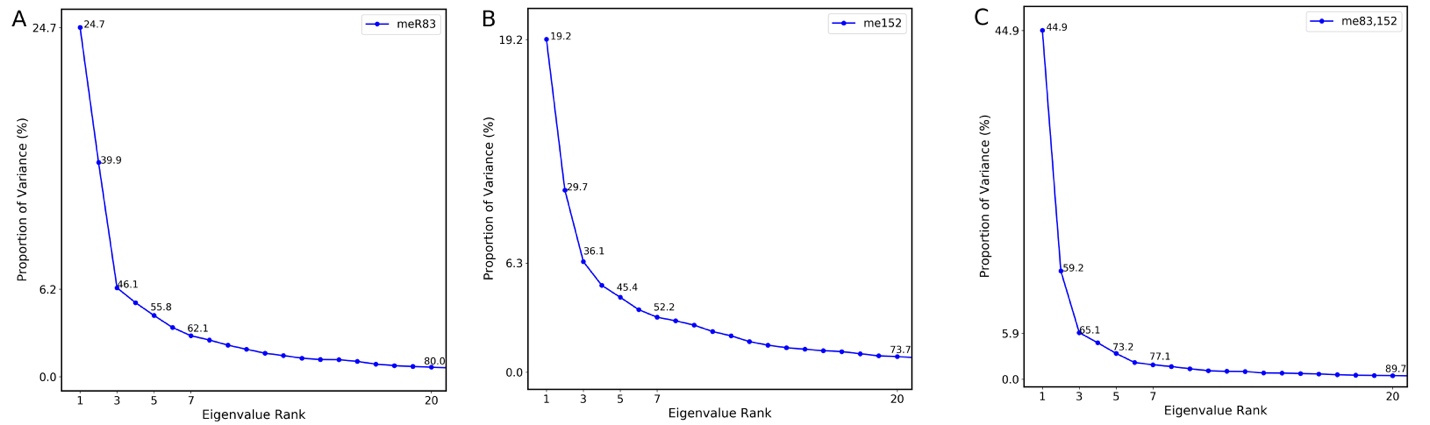
**

**Scree Plot.** Scree plot for principal component analysis on the MD data of DNA pol β complex (A) meR83, (B) meR152, and (C) meR83,152.
