# Supplementary material for: Deciphering the structural consequences of R83 and R152 methylation on DNA polymerase β using molecular modeling
Source: PLoS One. 2025 Mar 12;20(3):e0318614. doi: 10.1371/journal.pone.0318614 (PMC11902276; doi:10.1371/journal.pone.0318614)
Supplement: S6 Fig — DNA polymerase β each residue contribution to PC1 and PC2 of (A) meR83, (B)meR152, and (C) me83,152. The data are colored according to domain/subdomain color as shown in Fig 1A (in the main text). (DOCX) [file pone.0318614.s006.docx]

**S6 Fig.**

**
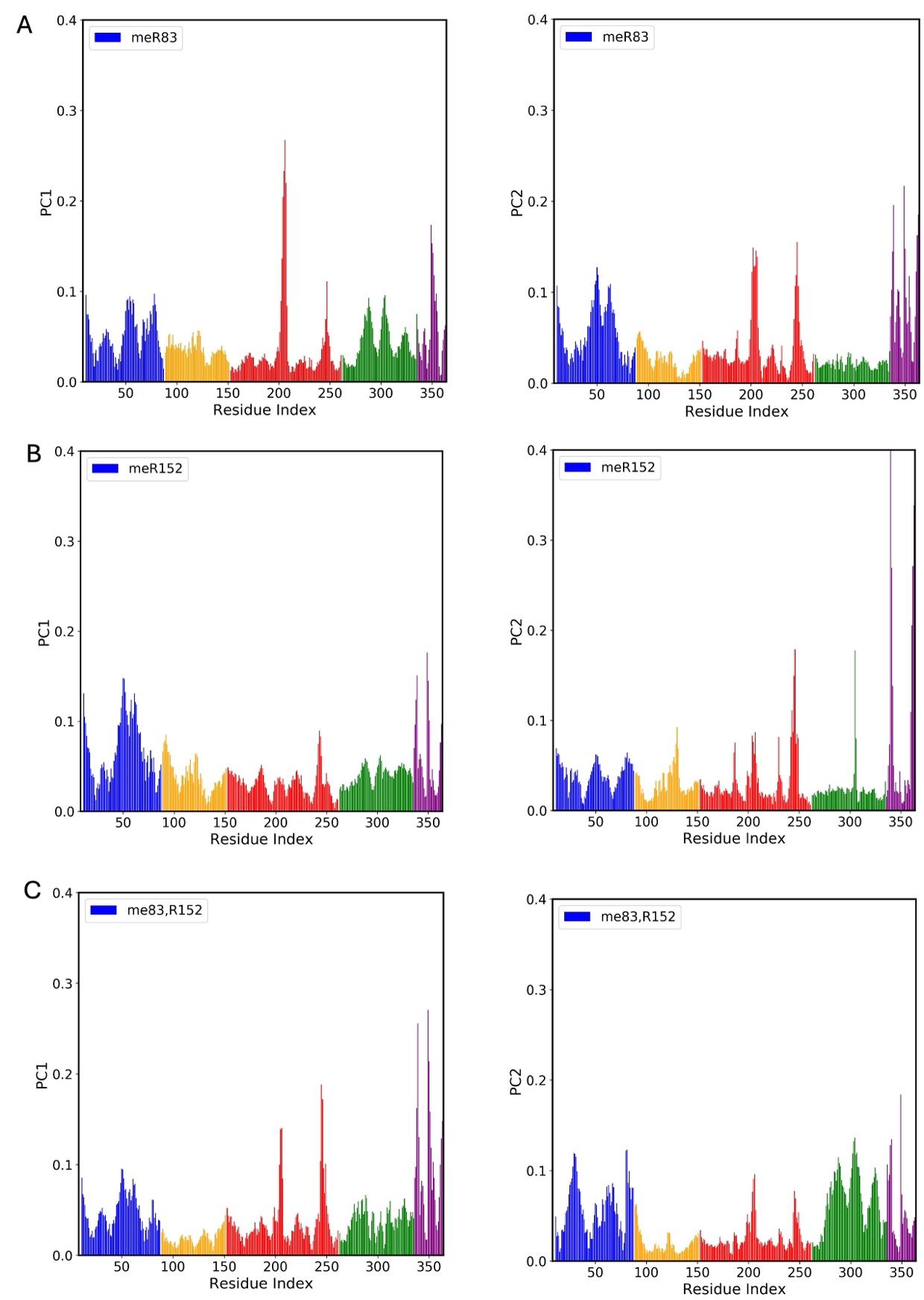
**

**Each residue contribution to Principal Component 1 (PC1) and Principal Component 2.** DNA polymerase β each residue contribution to PC1 and PC2 of (A) meR83, (B)meR152, and (C) me83,152. The data are colored according to domain/subdomain color as shown in Figure 1A (in the main text).
