# Supplementary material for: Deciphering the structural consequences of R83 and R152 methylation on DNA polymerase β using molecular modeling
Source: PLoS One. 2025 Mar 12;20(3):e0318614. doi: 10.1371/journal.pone.0318614 (PMC11902276; doi:10.1371/journal.pone.0318614)
Supplement: S7 Fig — Schematic representation of salt -bridges formed between the methylated residues and different domains and sub-domains shown on the DNA pol β structure (A) meR83,152 without DNA, and (B) meR83, 152 with DNA. Residues form salt bridges, as shown in stick representations. (DOCX) [file pone.0318614.s007.docx]

**S7 Fig.**

**
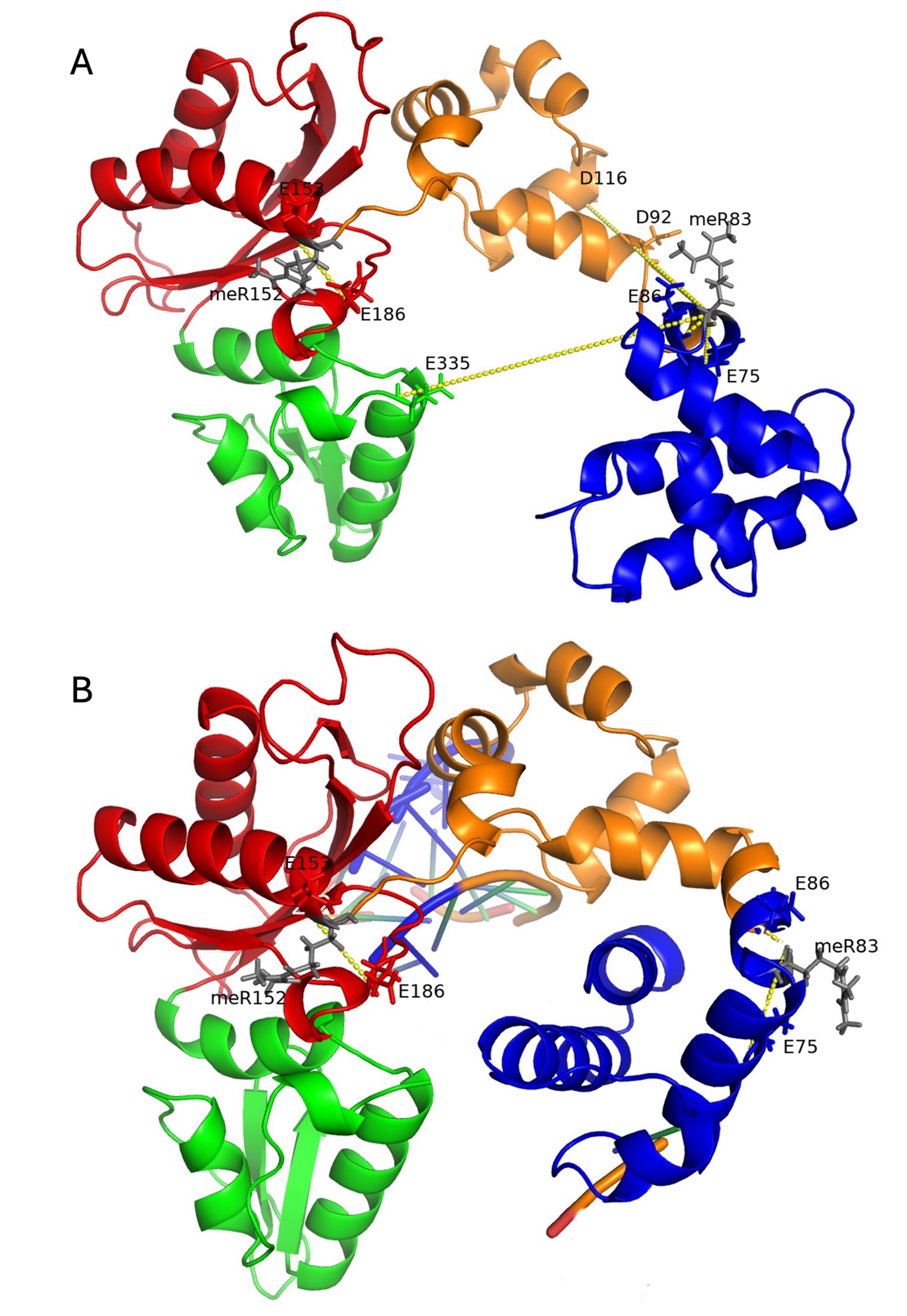
**

**Schematic representation Salt-bridges.** Schematic representation of salt -bridges formed between the methylated residues and different domains and sub-domains shown on the DNA pol β structure (A) meR83,152 without DNA, and (B) meR83, 152 with DNA. Residues form salt bridges, as shown in stick representations.
