# Supplementary material for: Deciphering the structural consequences of R83 and R152 methylation on DNA polymerase β using molecular modeling
Source: PLoS One. 2025 Mar 12;20(3):e0318614. doi: 10.1371/journal.pone.0318614 (PMC11902276; doi:10.1371/journal.pone.0318614)
Supplement: S8 Fig — Perturbation response analysis identifies the highly influential and sensitive residues that likely propagate allosteric signals in all three methylated systems. The effector provides information about the influence or effectiveness of a particular residue in transmitting signals when subjected to unit perturbation, while the sensor provides information about the sensitivity of a given residue to those signals. The effector signal of (A) WT and (B) meR83,152. The sensor signal of (C) WT and (D) meR83, 152. (DOCX) [file pone.0318614.s008.docx]

**S8 Fig.**

**
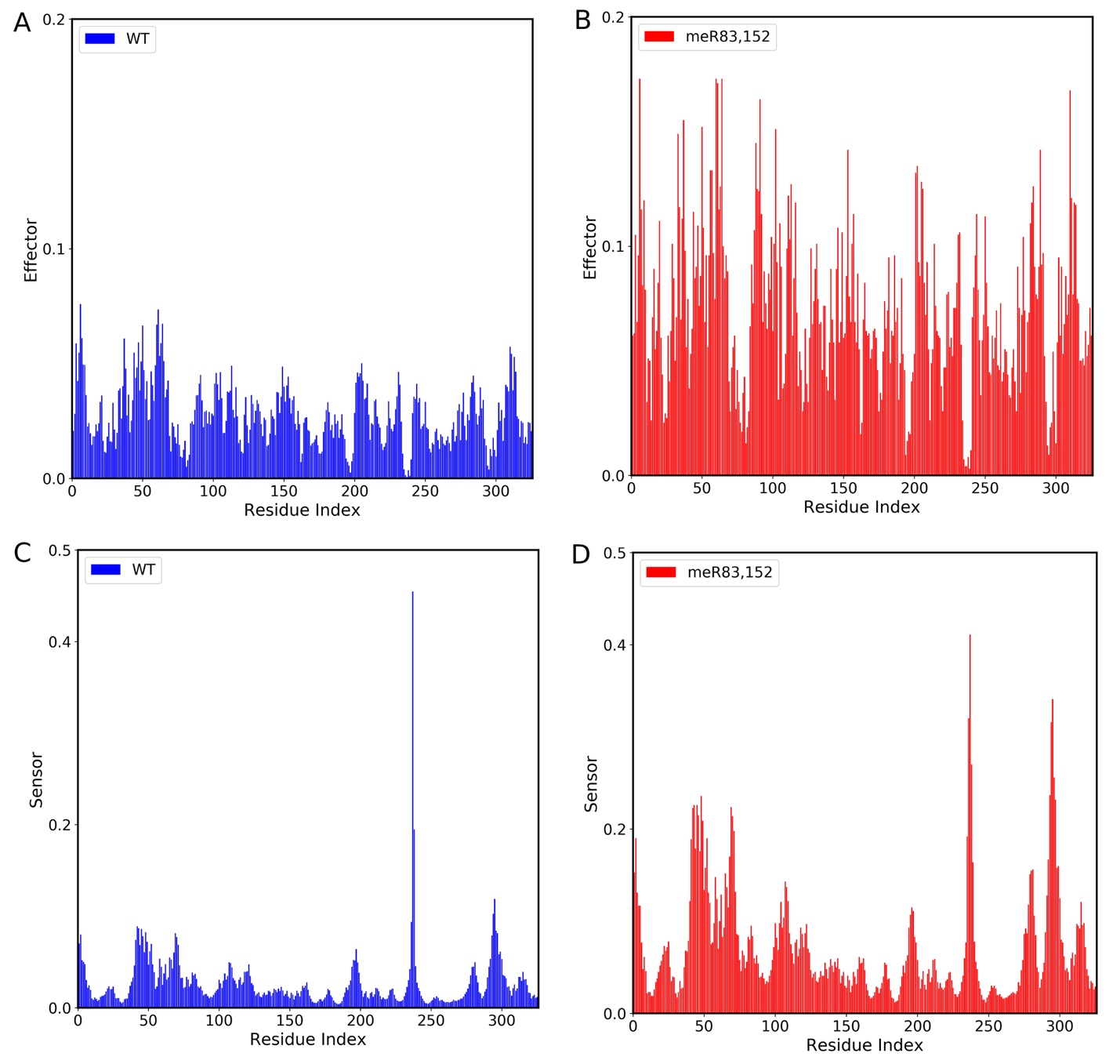
**

**Perturbation Response Analysis.** Perturbation response analysis identifies the highly influential and sensitive residues that likely propagate allosteric signals in all three methylated systems. The effector provides information about the influence or effectiveness of a particular residue in transmitting signals when subjected to unit perturbation, while the sensor provides information about the sensitivity of a given residue to those signals. The effector signal of (A) WT and (B) meR83,152. The sensor signal of (C) WT and (D) meR83, 152.
