# Supplementary material for: Deciphering the structural consequences of R83 and R152 methylation on DNA polymerase β using molecular modeling
Source: PLoS One. 2025 Mar 12;20(3):e0318614. doi: 10.1371/journal.pone.0318614 (PMC11902276; doi:10.1371/journal.pone.0318614)
Supplement: S1 Table — The donor-acceptor distance between S44 and E335 for all systems. (DOCX) [file pone.0318614.s009.docx]

**S1 Tab.**

| System | Average dist. (Å) | St. Dev. (Å) |
| --- | --- | --- |
| WT | 6.18 | 2.10 |
| meR83 | 5.98 | 2.0 |
| meR152 | 6.45 | 1.9 |
| meR83,152 | 7.22 | 2.22 |

**Donor-Acceptor Distance.** The donor-acceptor distance between S44 and E335 for all systems.
